# The Health Halo Trend in UK Television Food Advertising Viewed by Children: The Rise of Implicit and Explicit Health Messaging in the Promotion of Unhealthy Foods

**DOI:** 10.3390/ijerph15030560

**Published:** 2018-03-20

**Authors:** Rosa Whalen, Joanne Harrold, Simon Child, Jason Halford, Emma Boyland

**Affiliations:** 1 Department of Psychological Sciences, University of Liverpool, Liverpool L69 7ZX, UK; harrold@liverpool.ac.uk (J.H.); jasonh@liverpool.ac.uk (J.H.); eboyland@liverpool.ac.uk (E.B.); 2 Cambridge Assessment, Cambridge CB1 2EU, UK; Child.S@cambridgeassessment.org.uk

**Keywords:** advertising, television, food marketing, childhood obesity

## Abstract

Monitoring the creative content within food marketing to children is strongly advocated by public health authorities, but few studies address the prevalence of health-related messaging in television adverts. Food and beverage adverts (*n* = 18,888 in 2008, *n* = 6664 in 2010) from UK television channels popular with children were coded and analyzed. Physical-activity depiction displayed an 18.8 percentage point increase from 2008 (4.4%) to 2010 (23.2%). Of the food adverts containing physical-activity depiction in 2010, 81.1% were for non-core foods. The appearance of health claims in food adverts in 2010 increased 4.1 percentage points from 2008 levels (20.7% to 24.8%) where the majority of food adverts featuring health and nutrition claims were for non-core foods (58.3%). Health-related (e.g., health/nutrition, weight loss/diet) appeals were used in 17.1% of food adverts during peak child-viewing times, rising to 33.0% of adverts shown on dedicated children’s channels in 2010. Implicit (physical activity) and explicit (health claims) health messages are increasingly prevalent in UK television food advertising viewed by children, and are frequently used to promote unhealthy foods. Policy makers in the UK should consider amendments to the existing statutory approach in order to address this issue.

## 1. Introduction

Whilst childhood obesity remains a significant public health challenge, children’s exposure to obesogenic food marketing presents a clear opportunity for intervention. Although few countries have effectively dealt with the issue [1] in the UK, Ofcom (the broadcast regulator) have imposed media-specific statutory regulation with the aim of reducing exposure for children under the age of 16 years to the advertising of foods high in saturated fats, sugars and/or salt (HFSS) on television [2]. The regulations, phased in during 2007–2009, banned HFSS adverts on dedicated children’s channels and restricted the broadcast of such adverts around programmes of appeal to 4–15 year olds on any channel [3].

An expansive global evidence base quantifies the frequency of television food adverts, where studies confirm that food adverts remain strongly biased toward less healthy options [4,5,6] even where regulations do exist [7]. Comparatively few studies examine the creative content of food advertising which plays a critical role in its persuasive effect [8]. The World Health Organisation calls on governments to introduce regulation that “has the ability to reduce the power of marketing by targeting the use of specific techniques which have a particularly powerful effect, a disproportionate influence, or to which children are particularly vulnerable” [9] (p. 20). Both legislative approaches (UK Ofcom regulations) [3] and voluntary initiatives (CFBAI) [10] purport to target the content of television food adverts to reduce their persuasive power or shift the mix of foods advertised to children under age 12.

In the context of greater marketing regulation, food manufacturers increasingly aim to position their products in the context of a ‘healthy and balanced diet’ within television adverts and wider marketing. The International Food and Beverage Alliance pledges to “raise awareness on balanced diets and increased levels of physical activity” [11] and the Food and Drink Federation states that marketing messages should communicate “healthy dietary habits and physical activity” [12]. Marketers assert that this encourages children to maintain healthy lifestyles and some evidence does support the potential of nutritional messaging, which may positively influence children’s short term consumption behaviours [13]. Other research demonstrates that health referencing used in some circumstances may lead to the misinterpretation of foods. One study reported that fast food restaurants leveraged healthy foods to promote unhealthy children’s fast food meal options [14], and this approach has been referred to as a “health halo” effect [15].

The healthhalo effect involves cognitive bias where an explicit claim about single health quality (e.g., “contains essential nutrients”) or an implicit reference within the marketing (e.g., physical activity depiction) gives rise to more positive impression of other, non-claimed qualities [15]. For example, researchers found that using the label “fruit sugar” instead of “sugar” increased the perceived healthiness of breakfast cereals [15] indicating that a less-healthful food may be considered as healthy based on the package claims. Both explicit and implicit health-related messages may alter perceptions of food products in similar ways. A recent experimental study explored the specific impact of 5–6 and 10–11 years old children’s perceptions of a television advert for a sugar-laden cereal depicting physical activity, compared with an advert for the same product without physical activity being referenced [16]. It found that the sugary-cereal advert containing a physical activity reference led children to believe that the cereal was a healthier option and failed to encourage engagement in physical activity depicted within the advert or more general exercise. Australian children were also more likely to choose energy-dense nutrient poor foods when packaging featured nutrient content claims [17]. Recent experimental data in children aged 7–11 has confirmed health halo effects (using nutrition and physical activity messages) in child-directed adverts for nutrient poor food and drinks [18]. It was reported that children viewing the health halo adverts rated the products in those adverts as significantly healthier than those children who saw other adverts for the same products without the health messaging.

This study aims to build on existing research quantifying the creative content of food and beverage (hereafter referred to as ‘food’) advertising, with a specific focus on the use of health-related messaging as part of a health halo effect. The study will explore the depictions of physical activity and use of health-claims and themes in food advertising (for unhealthy, healthy and miscellaneous products) across a range of channels popular with children, as well as exploring differences across channel types and between peak and non-peak children’s viewing times. By re-examining broadcast data from 2008 [19] and coding broadcast data from 2010 (after the full implementation of the regulations), health-related messages in food advertising are measured over time to identify any changes (as may be expected following the legislative action). Such monitoring is recommended by both researchers [20] and public health authorities [8,9], and these data could allow policy-makers to determine what areas of food marketing may warrant further regulation to restrict misleading health-related messaging.

## 2. Materials and Methods

Television recordings were made on one week day (Tuesday or Thursday) and one weekend day (Saturday or Sunday) over 12 months of 2008 and six months of 2010 (February, April, June, August, October and December). This resulted in 5233.5 h of television from 2008 and 1931.5 h from 2010 (missing data due to recording errors). Food and beverage adverts (*n* = 18,888 in 2008, *n* = 6664 in 2010) from UK television channels popular with children were coded and analysed. Six months of 2010 television recording is likely to be highly representative of the full 12 months, we found a less than a 1% change in proportions of food types advertised between the 12 months published data of 2008 compared to six months [21].

The channels monitored for the current study were the same as those used by Boyland et al. [7] in order to enable comparisons to be made between the two sets of data. These were Nickelodeon, Cartoon Network, Boomerang, and CiTV (children’s channels); Sky Sports 1 (sports channel); ITV1, Channel Four, Channel Five, Sky1 and E4 (family channels) and 4Music, Smash Hits, and MTV (music channels). This list reflects those commercial channels with the greatest viewing share for children aged 4–15 years and those reported to be popular with 5–16 year olds [22]. In addition, the 2008 sample monitored children’s channel Jetix, which was taken off air between the two recording times. On each test day, television was recorded from 06:00–22:00 (16 h), with the exception of CiTV which only broadcasts for 12 h (6:00–18:00).

Coding across channel types, food types and children’s viewing times was performed in line with previous research with adherence to an established coding scheme used in this area [7,23]. Specific to this study, previously published data from 2008 [7] was re-examined and new data [21] coded to examine health-related content of food adverts. Food/beverage items were categorised into one of 29 food groups of which all were exclusively core(healthy), non-core (unhealthy) or miscellaneous items (see Table A1).

Any physical activity depiction, defined as the visual presentation of one or more individuals engaged in exercise beyond walking (not in background), was coded. In addition, health claims were coded across 15 categories including “no added sugar”, “provides essential nutrients (e.g., calcium, vitamins, antioxidants)”, and “part of your five a day”. Both verbal and textual health messages were coded and where more than one claim was made, the main claim was coded (if more than one main claim was found, the first mentioned was coded). The primary persuasive appeal used in each advertisement was also coded in accordance with the pre-established criteria. Categories included health-related (e.g., health/nutrition, weight loss/diet) and non-health-related appeals (e.g., enjoyment/satisfaction, fun) so that shifts towards or away from the use of health-related appeals could be identified (see Table A2). If more than one persuasive appeal was used the most dominant appeal was coded. In the event that this was not clear, the appeal featuring first was used.

To assess coding reliability between researchers, a random two hour sample of television was coded by both researchers and compared for consistency (as conducted for food categories [21]). For physical activity depiction, agreement was 83.3% between coders with moderate agreement between coders (*κ* = 0.571). For health claims, agreement was 100% between coders (*κ* = 1.000). For primary persuasive appeal, agreement was 66% between coders (*κ* = 0.571).

## 3. Results 

### 3.1. Physical Activity Depiction

Overall, depiction of physical activity in television food adverts displayed an 18.8 percentage point increase (hereafter referred to as percent point) from 2008 (4.4%) to 2010 (23.2%). Increases were seen across all channels, on sports (+12.5, from 6.9% in 2008 to 19.4% in 2010), family (+15.4, from 3.7% in 2008 to 19.1% in 2010) and music (+11.8, 3.3% in 2008 to 15.1% in 2010) channels, but the largest percent point increase was seen on children’s channels from 5.2% in 2008 to 34.7% in 2010 (+29.5). During peak children’s viewing times, the frequency of physical activity depiction increased 15.2 percent points, from 5.1% in 2008 to 20.3% in 2010.

Of the food adverts containing physical activity depiction in 2010, 81.1% were for non-core foods. Common examples from this sample include adverts for food brands McDonalds, Robinson’s (sugar sweetened drinks) and Weetos (high sugar/low fibre breakfast cereal). See Table 1 for a breakdown of physical activity depiction in core, non-core and miscellaneous food adverts across the whole sample and by channel type, including how this has changed over time (2008 to 2010).

### 3.2. Health Claims

Overall, the appearance of health claims in food adverts in 2010 had increased 4.1 percent points from 2008 levels (20.7% to 24.8%). The largest increase was seen on children’s channels (+8.8), from 22.2% in 2008 to 31% in 2010. Increases were also seen across family (+2.9, from 21.4% in 2008 to 24.3% in 2010) and music (+2.2, from 21.2% in 2008 to 23.4% in 2010) channels. In 2010, the majority of food adverts featuring health and nutrition claims were for non-core foods (58.3%). Of these, the most frequent non-core food adverts featuring health claims in 2010 were for full cream milk products (25.1%), high fat spreads (14.1%) and high sugar and/or low fibre breakfast cereals (12.2%). Of all food adverts including health claims, 26.1% were for core food items in 2010 and within these, 51.1% were for low fat dairy products and 16.6% for low sugar and high fibre breakfast cereals. Of the food adverts containing health claims in 2010, 15.5% were promoting miscellaneous food items, the majority of which were for infant milk formula (58.6%). 

On dedicated children’s channels, of the food adverts featuring health claims, 64.1% were for non-core foods. The most frequent non-core food adverts containing a health claim on children’s channels were McDonald’s Happy Meal adverts (35.6%) for meal bundles as described above. See Table 2 for health claims across channel type and change from 2008. The most frequently used health claim in 2010 was “contains essential nutrients…”, Of food adverts featuring this health claim, yogurts and yogurt drinks were the most common (40.9%) with claims such as “contains calcium and vitamin D which helps build strong bones”. Examples of non-core food adverts using this claim include a promotion for McDonald’s Happy Meal containing a milk drink: “milk contains calcium which is great for your teeth”. Consistent with the overall data, on dedicated children’s channels (see Table 2), the most heavily used health claim in the 2010 sample was “contains essential nutrients” at 39.7%. Of these 62.9% were for yogurt/yogurt drinks with reference to calcium.

### 3.3. Primary Persuasive Appeal

In 2010, health-related (e.g., health/nutrition, weight loss/diet) appeals were used in 17.1% of food adverts during peak child viewing times, an increase of 1.9 percent points from 14.9% in 2008. Non-health related persuasive appeals (see Table A2) comprised 83.0% in 2010. On dedicated children’s channels in 2010, health-related appeals during peak children’s viewing times were used in 33.0% of adverts, an increase of 15.9 percent points compared to the 2010 sample and an increase of 24.4 percent points from 2008.

## 4. Discussion

The current study systematically monitored television food advertising content in two broadcast samples (2008 and 2010) to examine use of health-related messages in television food advertising over time, as prioritised by World Health Organisation recommendations [24]. Compared with 2008, food adverts broadcast in 2010 were more likely to feature physical activity depiction and health claims, with the largest increases found for advertising on dedicated children’s channels. Crucially, overall in 2010, both physical activity depiction and health claims were most commonly featured within adverts for non-core, unhealthy foods. Health-related appeals were the most heavily used persuasive appeal across all food adverts in 2010, and constituted one third of appeals used on dedicated children’s channels in 2010. Together, these findings imply greater prominence of implicit (physical activity) and explicit (health claims) health-related messages in UK television food advertising.

Physical activity depiction within food adverts increased almost 20 percent points between 2008 and 2010, indicating that marketers are keen to align themselves with consumers’ interest of health by constructing an association with exercise. Results reported here are consistent with increases observed in a US study, where in 2009 physical activity was depicted in 6.6% of food adverts, increasing to 20.1% in 2013 [25]. Notably, the current research found the largest increase of physical activity on dedicated children’s channels; an increase of almost 30 percent points from 2008 to 2010. Debate surrounds this trend, which is often celebrated by the food industry for encouraging children to get fit [26]. Indeed, research shows that children as young as 5 years old associate physical activity with health [27]. However, these data found that the majority of food adverts depicting physical activity were promoting unhealthy foods, and this was almost exclusively the case on dedicated children’s channels. This is a concern as recent experimental research shows that physical activity depiction in unhealthy food marketing skews children’s perceptions of the healthfulness of foods and affects their appeal [25] thus, this marketing strategy may be detrimental to children’s understanding and ability to make informed food-related decisions. As children’s food knowledge has been found to impact their diet [8,28], research must continue to quantify this pattern over time and further investigate the implications of these marketing trends on children’s food choices. 

There was an increase in health and nutrition claims found in television food adverts from 2008 to 2010, mirroring increases found on food and beverage packaging in UK supermarkets [29]. The frequency of adverts featuring health claims was highest on children’s channels compared to all other channel types, in line with previous UK research [30]. The majority of health claims recorded in the present study promoted non-core foods (with this proportion having risen over time). On children’s channels, the most frequently used health claim was “contains essential nutrients” and an increase was observed in the use of “part of your five a day”, however it was found that these adverts were almost exclusively for McDonald’s Happy Meals. Use of the common traffic light label on such non-core food product packaging and advertising might be useful here to inform individuals about nutrients and food items that are less healthy. Additionally, regulatory agencies may wish to develop stricter regulations for nutrient content claims due to their prevalence and impact, particularly when the health association is unfounded. 

In the UK, Ofcom brought regulations into full effect in 2009 to ban HFSS adverts on dedicated children’s channels and restrict the broadcast of such adverts around programmes of appeal to 4–15 year olds on any channel [3]. However, data from the present study and published data [21] demonstrate that non-core foods are still being broadcast on children’s channels. This is likely to be in part due to differences in how advertised products are categorised by the nutrient profiling system used by the broadcast regulator and the standard coding framework used in this study (and in many other published studies of this kind [7,23]). Advertisements by food companies that produce, sell and promote largely unhealthy foods (e.g., fast food brands) were categorised here as non-core even if food product, as presented, passed the UK Ofcom nutrient profiling restrictions. As an example of where this is likely to lead to categorisation differences, McDonalds have been found to increasingly portray meal bundles with the healthier constituents (e.g., fish fingers, a fruit bag and a bottle of water rather than a burger, fries and a soft drink [31]). Adverts of this nature are still permitted to be broadcast to children but research has demonstrated that exposure increases children’s liking of fast food and does not lead them to make healthier choices [31]. Informed debate around this particular issue is warranted to determine whether legislation should restrict this practice. 

A limitation of this study is that the data were a random sample of adverts aired during the study periods, and as such may not be reflective of all adverts aired during these times. Moreover, due to differences in sample size (12 months of data for 2008 and six months for 2010) comparisons can only be made on the basis of proportional data (percentages and rates per hour) rather than number of occurrences. A further study limitation is that a narrow range of health imagery was coded. Future research should measure a broader variety of health-based cues, for example the use of fruit or vegetable imagery [32]. Finally, this study did not address children’s interpretation of the claims, and it is not clear if all health claims would impact children equally (for example, some health claims were likely to target parents).

## 5. Conclusions

Together, findings across physical activity depiction, health claim use and health-related primary persuasive appeal denote a substantial and increasing use of health-related messages and content within UK television food advertising on channels popular with children. Results demonstrate that nutritionally deficient foods are often paired with implicit and explicit references to health; with the heaviest use of such references on children’s channels and in the promotion of unhealthy foods. This marketing approach may mislead children in terms of their understanding of nutrition and lead to judgement errors with regard to healthy food choices. Researchers should explore further the impact of health messages used in conjunction with unhealthy foods on children’s food attitudes and intake. Policy makers in the UK should consider amendments to the existing statutory approach [2] to address this issue. In the US, the CFBAI recognized that using physical activity to promote unhealthy foods was deceptive in 2013. However, more progress needs to be made to understand what policy makers can do to ensure that child-directed food advertising does not encourage children to misperceive the nutritional qualities of unhealthy foods.

## Figures and Tables

**Table 1:** Health-related message use in all adverts in 2010 (*n* = 6664) across core, non-core and miscellaneous food advertisements and comparison with the 2008 sample (*n* = 18,888).

Health-Related Message	Core (%)	Core % Change v 2008 (+/−)	Non-Core (%)	Non-Core % Change v 2008 +/−)	Miscellaneous (%)	Miscellaneous % Change v 2008 (+/−)
Physical activity (all channels)	11.8	−17.1	81.1	+15.6	7.1	+1.5
Physical activity (children’s channels)	9.8	−8.9	90.2	+8.9	-	-
Physical activity (sports channels)	5.4	−21.1	94.5	+0.4	-	-
Physical activity (family channels)	14.1	−12.4	75.7	+11.9	10.2	+0.5
Physical activity (music channels)	12.8	−38.8	74.4	+30.2	12.8	−7.6
Health Claims (all channels)	26.1	−9.0	58.3	−5.5	15.5	+14.4
Health Claims (children’s channels)	29.4	+8.8	64.1	−15.3	6.5	+6.5
Health Claims (sports channels)	26.0	−38.0	59.0	+22.6	15	+15.0
Health Claims (family channels)	26.1	−11.3	56.3	−4.5	17.6	+15.9
Health Claims (music channels)	21.9	−21.7	54.0	−0.5	24.1	+22.2

**Table 2:** The most heavily used health claims on dedicated children's channels in 2010, change from 2008 health claim use across all channels in 2010.

Health Claims (Dedicated Children’s Channels)	2010 (%)	% Change v 2008 (+/−)	2010 All Channels (%)
Contains essential nutrients e.g., calcium, vitamins, antioxidants	39.7	+29	33.4
Part of your five a day	28.9	+4.9	10.3
Organic	10.4	+8.8	5.0
Wholegrain	8.3	−11.8	7.8
Low fat	4.7	+2.8	9.8
No added sugar	3.7	−0.5	2.1
Contains only natural ingredients	3.1	−12.2	15.9
Contains fibre	0.6	+0.6	1.3
Is low calorie/light	0.6	−5.1	5.1

## Appendix A

**Table A1:** Core, non-core and miscellaneous food and beverage coding system [23].

Core and Healthy Food Categories
1 Breads (include high fibre, low fat crackers), rice, pasta and noodles
2 Low sugar and high fibre breakfast cereals (<20 g/100 g sugar and >5 g/100 g dietary fibre)
3 Fruits and fruit products without added sugar
4 Vegetables and vegetable products without added sugar
5 Low fat/reduced fat milk, yoghurt, custard (<3 g/100 g fat) and cheese (<15 g/100 g fat; includes 50% reduced fat cheddar, ricotta and cottage) and their alternatives (e.g., soy) (including probiotic drinks)
6 Meat and meat alternatives (not crumbed or battered) (includes fish, legumes, eggs and nuts and nut products, including peanut butter and excluding sugar coated or salted nuts)
7 Core foods combined (including frozen meals (<10 g/serve fat), soups (<2 g/100 g fat, excludes dehydrated), sandwiches, mixed salads and low fat savoury sauces (<10 g/100 g fat; includes pasta simmer sauces)
8 Baby foods (excluding milk formulae)
9 Bottled water (including mineral and soda water)
Non-core and unhealthy food categories
10 High sugar and/or low fibre breakfast cereals (>20 g/100 g or <5 g/100 g dietary fibre)
11 Crumbed or battered meat and meat alternatives (e.g., fish fingers) and high fat frozen meals (>10 g/serve fat)
12 Cakes, muffins, sweet biscuits, high fat savoury biscuits, pies and pastries
13 Snack foods, including chips, savoury crisps, extruded snacks, popcorn, snack bars, muesli bars, sugar sweetened fruit and vegetable products (such as jelly fruit cups, fruit straps) and sugar coated nuts
14 Fruit juice and fruit drinks
15 Frozen/fried potato products (excluding packet crisps)
16 Full cream milk, yoghurt, custard, dairy desserts (>3 g/100 g fat) and cheese (25% reduced fat and full fat varieties, and high salt cheese, including haloumi and feta) and their alternatives
17 Ice cream and iced confection
18 Chocolate and confectionery (including regular and sugar-free chewing gum and sugar)
19 Fast food restaurants/meals (include general pizza, burgers, ‘healthy’ alternatives from fast food restaurants)
20 High fat/sugar/salt spreads (includes yeast extracts, excludes peanut butter), oils, high fat savoury sauces (>10 g/100 fat), meal helpers (including stocks, tomato paste) and soups (>2 g/100 g fat tinned and all dehydrated)
21 Sugar sweetened drinks including soft drinks, cordials, electrolyte drinks and flavour additions e.g., Milo)
22 Alcohol
**Miscellaneous**
23 Vitamin and mineral supplements
24 Tea and coffee
25 Supermarkets—advertising mostly non-core foods
26 Supermarkets—advertising mostly core foods
27 Supermarkets—non-specified (generic supermarket ads or not clearly for core or non-core)
28 Baby and toddler milk formulae
29 Home food delivery services

**Table A2:** Primary persuasive appeals coding system [23].

Primary Persuasive Appeal
1 Quantity
2 Convenience
3 Taste
4 Health/Nutrition *
5 Energy
6 Price
7 Unique/New
8 Fun
9 General Superiority
10 Peer Status/Sex Appeal
11 Premium or Contest
12 Weight Loss/Diet *
13 Offers Choices/Options
14 Enjoyment/Satisfaction
15 Product Introduction
16 Corporate Information
17 Other

* = health-related primary persuasive appeal.
